# Robust effect of metabolic syndrome on major metabolic pathways in the myocardium

**DOI:** 10.1371/journal.pone.0225857

**Published:** 2019-12-02

**Authors:** Maryam Karimi, Vasile I. Pavlov, Olivia Ziegler, Nivedita Sriram, Se-Young Yoon, Vahid Agbortoko, Stoiana Alexandrova, John Asara, Frank W. Sellke, Michael Sturek, Jun Feng, Boian S. Alexandrov, Anny Usheva

**Affiliations:** 1 Department of Surgery, Rhode Island Hospital, The Warren Alpert Medical School, Brown University, Providence, RI, United States of America; 2 Icahn School of Medicine at Mount Sinai, New York, NY, United States of America; 3 Beth Israel Deaconess Medical Center, Harvard Medical School, Boston, MA, United States of America; 4 Indiana University, School of Medicine, Indianapolis, IN, United States of America; 5 Los Alamos National Laboratory, Los Alamos, NM, United States of America; Universidade de Mogi das Cruzes, BRAZIL

## Abstract

Although the high-fat-diet-induced metabolic syndrome (MetS) is a precursor of human cardiac pathology, the myocardial metabolic state in MetS is far from clear. The discrepancies in metabolite handling between human and small animal models and the difficulties inherent in obtaining human tissue complicate the identification of the myocardium-specific metabolic response in patients. Here we use the large animal model of swine that develops the hallmark criteria of human MetS. Our comparative metabolomics together with transcriptomics and computational nonnegative matrix factorization (NMF) interpretation of the data exposes significant decline in metabolites related to the fatty acid oxidation, glycolysis, and pentose phosphate pathway. Behind the reversal lies decreased expression of enzymes that operate in the pathways. We showed that diminished glycogen deposition is a metabolic signature of MetS in the pig myocardium. The depletion of glycogen arises from disbalance in expression of genes that break down and synthesize glycogen. We show robust acetoacetate accumulation and activated expression of key enzymes in ketone body formation, catabolism and transporters, suggesting a shift in fuel utilization in MetS. A contrasting enrichment in O-GlcNAcylated proteins uncovers hexosamine pathway and O-GlcNAcase (OGA) expression involvement in the myocardial response to MetS. Although the hexosamine biosynthetic pathway (HBP) activity and the availability of the UDP-GlcNAc substrate in the MetS myocardium is low, the level of O-GlcNacylated proteins is high as the O-GlcNacase is significantly diminished. Our data support the perception of transcriptionally driven myocardial alterations in expression of standard fatty acids, glucose metabolism, glycogen, and ketone body related enzymes and subsequent paucity of their metabolite products in MetS. This aberrant energy metabolism in the MetS myocardium provide insight into the pathogenesis of CVD in MetS.

## Introduction

Metabolic syndrome (MetS) has been established as a precursor of cardiac pathology and has been related to alterations in cardiac energy metabolism. While the role of metabolism in cardiac function has been overshadowed for the last 20 years with the advent and popularity of genetic analysis, there is now a growing appreciation for metabolic processes implicated in myocardial energy substrate availability that may contribute to the progression of cardiac pathology.

MetS is a cluster of metabolic conditions including obesity, hyperglycemia, insulin resistance, hypertriglyceridemia, elevated plasma LDL, high blood pressure, and endothelial dysfunction that put the patients at risk for heart disease and diabetes [1]. Given the multifaceted nature of MetS it is unlikely that single molecule biomarkers or dysregulation can adequately capture or prognosticate its development [2]. As such, recent investigations focus on applying quantitative methods for simultaneous screening of a large set of metabolites to characterize the intracellular metabolic milieu of MetS.

Targeted metabolomics data on a set of polar metabolites in the blood plasma, mainly some amino acids and their derivatives, was recently reported in patients with obesity and MetS [3, 4]. Although metabolomics analyses of blood and other body fluids provide valuable results, it is advantageous to analyze the tissue level changes in the myocardium given its unique metabolism. Currently, there is active investigation into the role of metabolic changes in the human myocardium, though this is hindered by the difficulty inherent in obtaining human tissue [5]. Here we use the large animal model of swine that develops the hallmark criteria of human MetS to overcome the discrepancies in metabolite handling between human and small animal models and the difficulties inherent in obtaining human tissue. The Yorkshire swine have been demonstrated to develop MetS that is nearly identical to humans, in a relatively short period of time when fed a hypercaloric, hypercholesterolemic diet [6, 7].

Here we use our high-fat-diet-induced MetS and lean-diet control (LD) Yorkshire swine to establish the metabolic picture of the MetS identity in the myocardium by applying a combined experimental and unsupervised nonnegative matrix factorization (NMF) computational approach, concordant metabolomics integration with transcription and physiological data. Overall, our data and analysis provide a picture of the MetS identity in the myocardium and do represent a major step forward uncovering targets for intervention and control of the myocardial disease processes in MetS.

## Results

### The impact of hypercaloric, hypercholesterolemic diet on myocardial MetS risk factors

**Fig 1:**
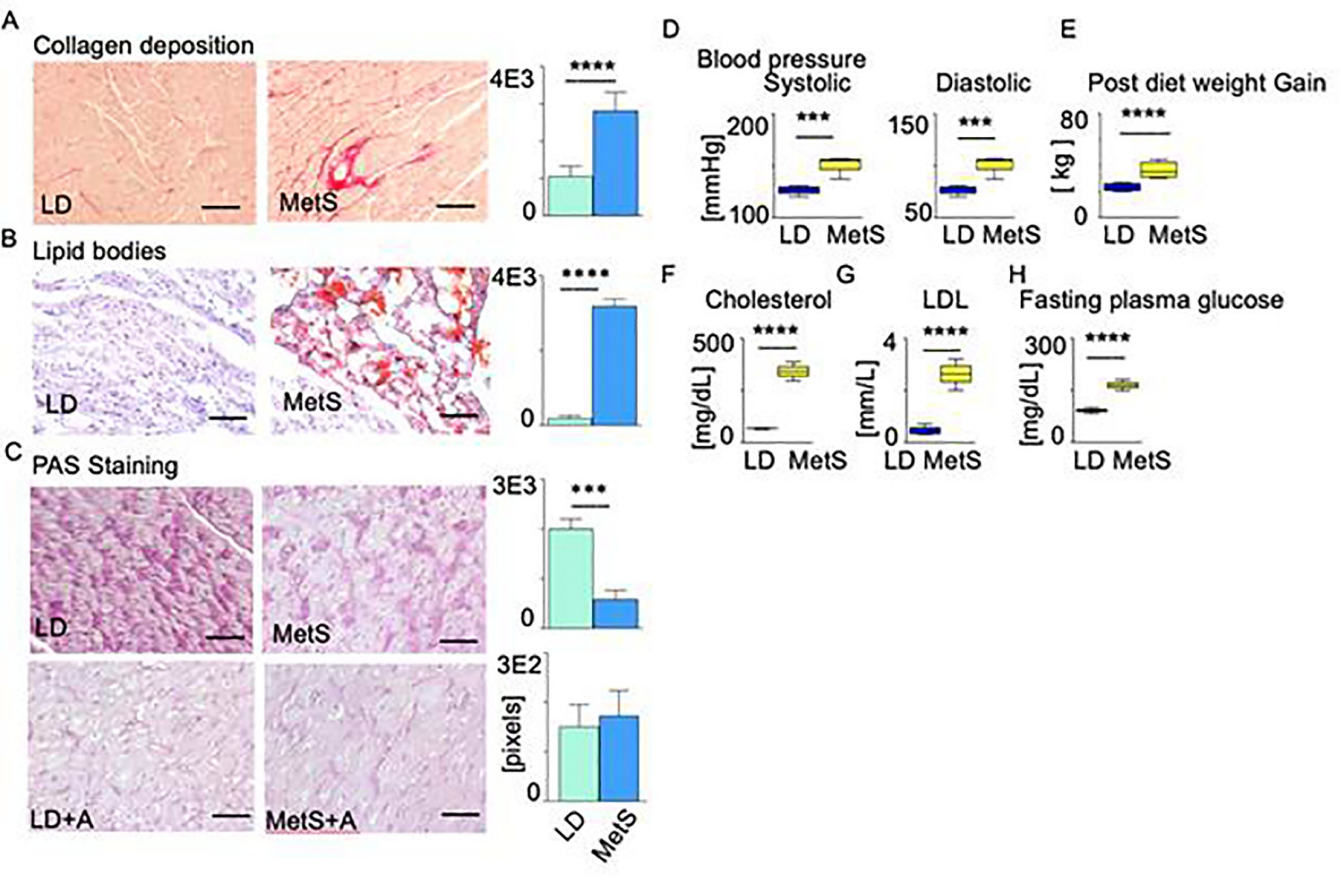
Impact of diet on metabolic syndrome risk factors in swine. MetS and LD swine are histologically compared by using paraffin embedded or cryo-preserved myocardial left ventricular tissue. Data are represented as means ± SD (n = 4 MetS, n = 4 LD). Representative images of myocardial tissue are shown at x20 high-power field, **p<0.01, ***p<0.001, ****p<0.0001 by Mann-Whitney U test. Scale bars: 20 μm (A, B, C). (A) Collagen deposition was evaluated by picrosirius red staining. The bar diagram on the right presents collagen staining (red) intensity in pixels. (B) Lipid oil red O staining for neutral triglycerides and lipids accumulation in frozen tissue sections. The bars diagram on the right presents the positive (red) staining intensity in the MetS (n = 6) and LD tissues (n = 6) in pixels (p = 0.0001). C) LD and MetS tissue sections are stained with PAS. LD tissue sections are treated with Amylase before PAS staining (LD+A, MetS+A panel). The difference in the PAS staining intensity before and after amylase treatment is used to calculate the glycogen content. The bar diagrams presents glycogen deposition (relative units) in the MetS and LD tissues (p = 0.001). (D) Systolic and diastolic blood pressure [mmHg] in MetS and LD pigs one week before surgery (n = 6 MetS and n = 6 LD, p = 0.0001). (E), Post diets weigh gain in kg for LD and MetS (n = 6 MetS and n = 6 LD, p = 0.0001), (F) cholesterol (n = 6 MetS and n = 6 LD, p = 0.0001), (G) plasma LDL (n = 6 MetS and n = 6 LD, p = 0.0001), (H) fasting plasma glucose (n = 6 MetS and n = 6 LD, p = 0.0001). Data represent mean± SD. ***p<0.001, 2-tailed Student’s t test.

The high-fat-diet-induced MetS in our swine model is associated with altered myocardial structure, blood pressure, and myocardial metabolism (Fig 1). We registered that the left ventricular histological tissue sections obtained from four swine on a hypercaloric, hypercholesterolemic diet show presence of increased collagen deposition (Fig 1A, n = 4 MetS, n = 4 LD, p = 0.0001) and intracellular lipid bodies accumulation (Fig 1B; n = 4 MetS, n = 4 LD, p = 0.0001) together with significantly lower glycogen deposition (Fig 1C; n = 4 MetS, n = 4 LD, p = 0.001) compared to the swine on a control lean diet (LD). As shown in Fig 1D (n = 4 MetS, n = 4 LD, p = 0.001), the MetS swine have significantly elevated systolic and diastolic blood pressure and post diet weight gain (Fig 1E; n = 4 MetS, n = 4 LD, p = 0.0001) compared to the LD animals. The animals in the MetS group developed key components of metabolic syndrome including elevated dyslipidemia (Fig 1F, n = 4 MetS, n = 4 LD, p = 0.0001), plasma LDL (Fig 1G, n = 4 MetS, n = 4 LD, p = 0.0001), and fasting plasma glucose (Fig 1H, n = 4 MetS, n = 4 LD, p = 0.0001).

Together, the phenotype observed in MetS pigs meet the five MetS diagnostic criteria: obesity, elevated fasting blood sugar, high low-density lipoprotein (LDL) and triglyceride level, and increased blood pressure.

### Metabolomics analysis and extraction of metabolic signatures by unsupervised learning approach uncover a MetS-specific profile of polar metabolites

**Fig 2:**
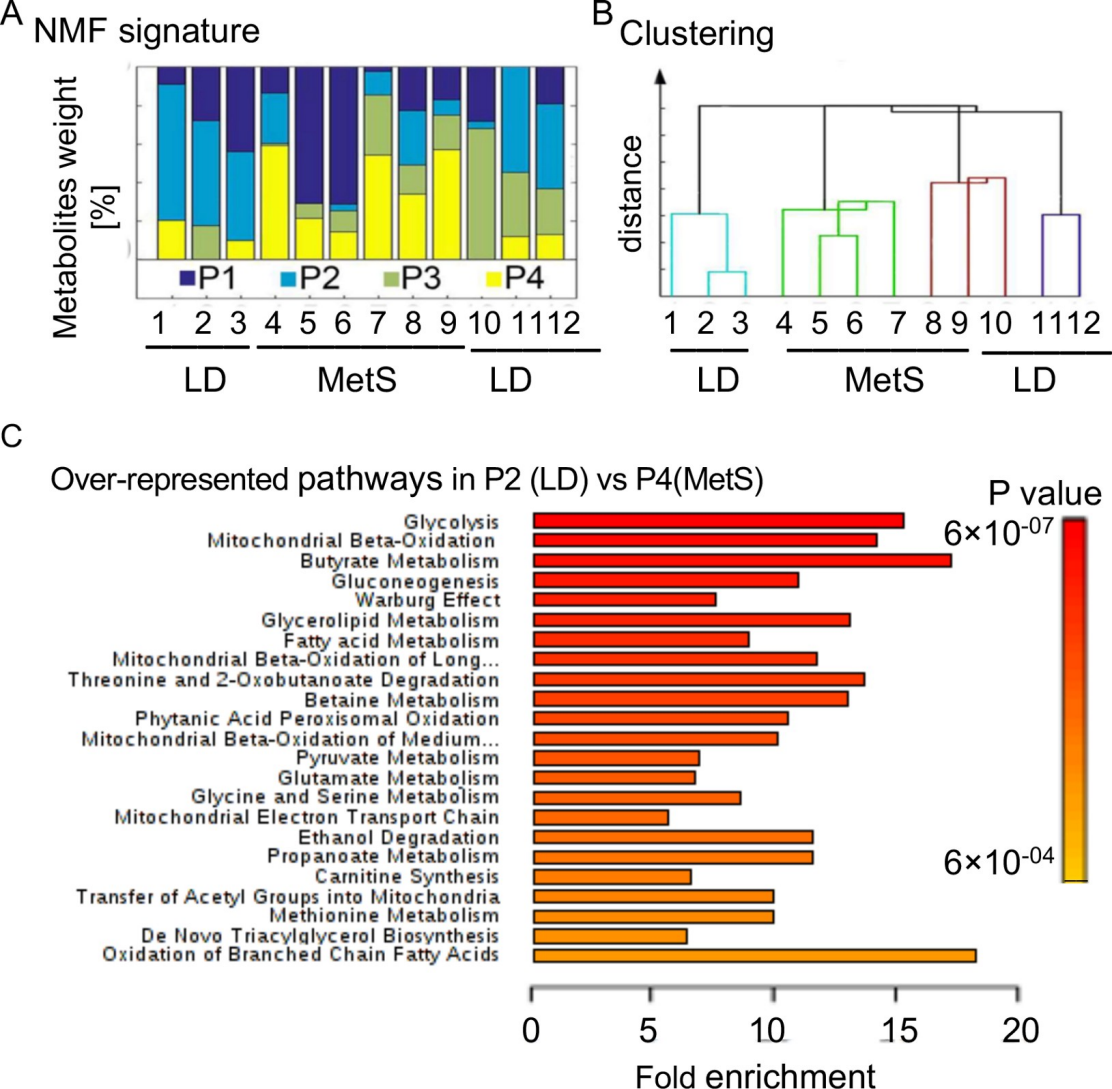
Metabolomic signatures of the myocardial response to diet are obtained by NMF. LC/MS-MS was applied to individually identify and measure polar metabolites (280 polar metabolites) in left ventricular cardiac tissue from Yorkshire intact male swine (MetS n = 6, LD n = 6). (A) Based on the LC/MS-MS derived 12 pig data sets of 280 polar metabolites, NMF extracted signatures of metabolic processes that clearly defer in MetS vs LD. The identity of the individual pigs is shown below the bars. The signatures P1, P2, P3, and P4 are shown in color below the bars. (B) Cluster dendrogram generated by unsupervised hierarchical clustering based on contributions of the four signatures identified by NMF to the metabolomics database of the 12 swine (cophenetic correlation coefficient 0.92). (C) Over-represented known pathways in the P2 (LD) signature vs. MetS signature (P4) are determined in MetaboAnalyst 4.0. Their p values are shown with the colored bar diagram on right.

We hypothesized that the high-fat-diet-induced MetS would produce specific metabolite alterations. Therefore, we extracted the polar metabolites fraction from the left ventricles of MetS and LD swine, then identified them and compared their content through liquid chromatography-tandem to mass spectrometry (LC-MS/MS) [8]. We analyzed individual tissue from 12 pigs: Yorkshire MetS (n = 6) and Yorkshire LD (n = 6) and identified 280 metabolites that are present in all samples at variable levels. To characterize the basic metabolite signatures from the MetS and LD datasets, we utilized an unsupervised learning approach based on NMF [9–11]. NMF extracted four “meta- metabolite” signatures (P1, P2, P3, P4), each including distinct combinations of the 280 metabolites from the 12 pigs (Fig 2A). The different weights of the four extracted signatures account for the variations in the 12 pig samples. Hierarchical clustering of the metabolites by NMF weights of the four signatures shows that the six MetS pigs are clustered together, while five of the LD pigs are clustered separately (Fig 2B). The clustering of the weights identifies P2 as the predominant signature in LD pigs and P4 as the predominant in MetS pigs. MetaboAnalyst 4.0 analyses of the P2 and P4 metabolites constituents show that the overrepresented processes underlying the P2 signature in LD are related to the glucose metabolism including glycolysis (p = 0.001) and glycogenolysis (p = 0.001) (Fig 2C). They are the first diminished processes that are statistically significant in the MetS signature, P4. Also overrepresented are processes related to the fatty acid metabolism (p = 0.009) and mitochondrial -oxidation of fatty acids (p = 0.04). Processes that are related to ketone bodies however, are overrepresented in P4 (p = 0.01). Each signature is characterized by a different metabolites content, and distinct combinations of these four signatures account for the variations in our 12 pig samples. The metabolites that are overrepresented in P2 vs P4 as identified by NMF are shown in S1 Table (supporting information).

Overall, the comparison of both signatures suggests for glycolysis, fatty acids, HBP (hexosamine biosynthetic pathway), glycogenolysis disbalance in MetS.

#### Metabolites composition of P2 and P4

**Fig 3:**
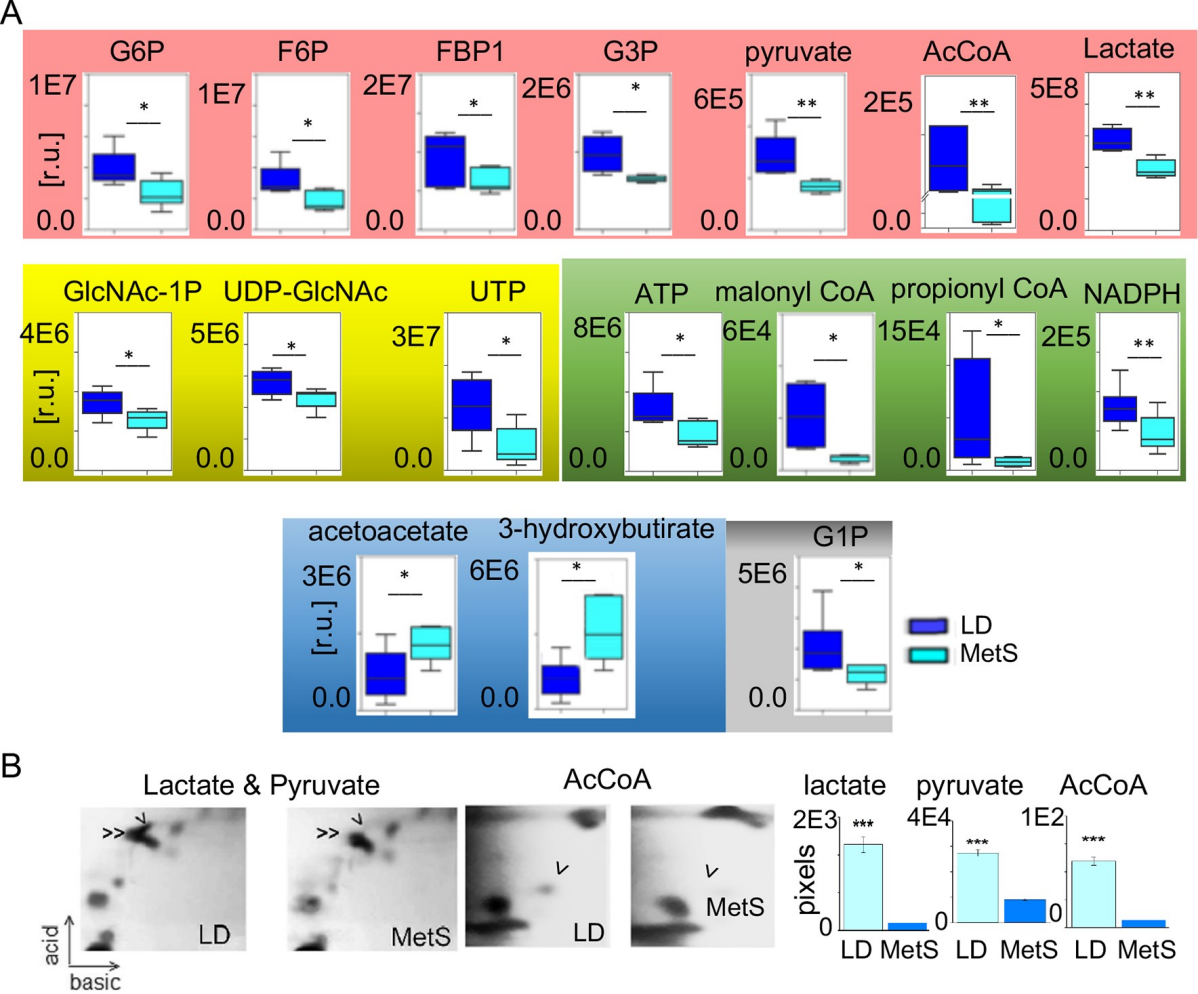
Key metabolic changes in the MetS heart. (A) LC/MS-MS data reveal significant alterations in content of metabolites in MetS vs. LD. The LC/MS-MS data are equalized based on protein content. GAPDH is used as an internal loading standard as well. The barrel diagrams show the individual metabolites content (relative units, r.u.) in LD (n = 6) and in MetS (n = 6). The identity of the metabolites is shown at the top of the diagrams. The metabolites that are related to glycolysis are highlighted in pink, hexosamine biosynthetic pathway in yellow, fatty acid oxidation related metabolites in green, ketone body family in blue, and glycogenolysis product in gray. Values are means ± SD. G6P, UTP (p = 0.01); acetyl-CoA (p = 0.006); NADPH (p = 0.009); F6P, FBP1, G3P, ATP, malonyl-CoA, acetoacetate, GlcNAc-1P (p = 0.02); pyruvate (0.004); 3-hydroxybutirate, G1P (p = 0.03); propionyl-CoA (0.05); lactate (p = 0.005). *p<0.05, **p<0.01, 2-tailed Student’s t-test. (B) 2d TLC separation of lactate and pyruvate in LD and MetS tissue: single arrow—lactate position, double arrow–pyruvate, AcCoA -single arrow. The bar diagram on the right shows the spot intensity (pixels) after background correction representing the average value of three independent extracts, each from 20 mg of tissue: MetS (n = 3), LD (n = 3). Data are presented as means ±SD. ***p<0.001, 2-tailed Student’s t-test.

We next compared P2 (LD) and P4 (MetS) contents of metabolites (Fig 3). The quantitative LC/MS-MS data (Fig 3A) derived from six MetS and six LD independent runs show a significant decrease in MetS vs LD of: glucose-6-phosphate (G6P) (p = 0.01); fructose-6-phosphate, (F6P) (p = 0.02) both of which are glycolysis entry metabolites, part of the HBP and UDP-GlcNAc precursors. Less abundant in MetS is fructose-l, 6-bisphosphate (FBP1) (p = 0.02), glycerol-3-phosphate (G3P) (p = 0.02) from the ATP reproductive glycolysis step. Pyruvate (p = 0.004) and lactate (p = 0.005), which represent glycolysis completion, are significantly reduced in content in P4 as well. In MetS, the LC/MS-MS data show a significant decrease of acetyl-CoA (p = 0.006), ATP (p = 0.02) and NADPH (p = 0.009) as well as malonyl-CoA (p = 0.02) and propionyl-CoA (p = 0.05). While glucose and fatty acid oxidation related metabolites are less presented in MetS, the abundance of acetoacetate (p = 0.02) and 3-hydroxybutirate (p = 0.03) from the ketone body family is significantly elevated vs LD. The glycogenolysis product G1P however is less abundant in the MetS signature (p = 0.03). The observations were further confirmed by two-dimensional thin layer chromatography (2d TLC). TLC analyses (Fig 3B) of extracts from four MetS and four LD pigs consistently show more than 3-fold decrease in lactate (n = 4, p <0.01, 4.31 ± 0.24 fold change), pyruvate (n = 4, p<0.01, 3.26 ± 0.51 fold change), and acetyl-CoA (n = 4, p<0.01, 3.16 ± 0.32 fold change) in MetS vs. LD.

The mass spectrometry-derived data expose diminished accumulation of metabolic intermediates related to glycolysis, hexosamine biosynthetic pathway, fatty acid oxidation, and glycogenolysis in MetS. Importantly, the ketone bodies representatives acetoacetate and 3- hydroxybutirate are more abundant in MetS than in LD suggesting increased availability of reliance on ketone bodies as a fuel in MetS.

#### mRNA expression levels of enzymes that are involved in glycolysis, HBP, fatty acids metabolism, glycogen accumulation, and ketogenesis

**Fig 4:**
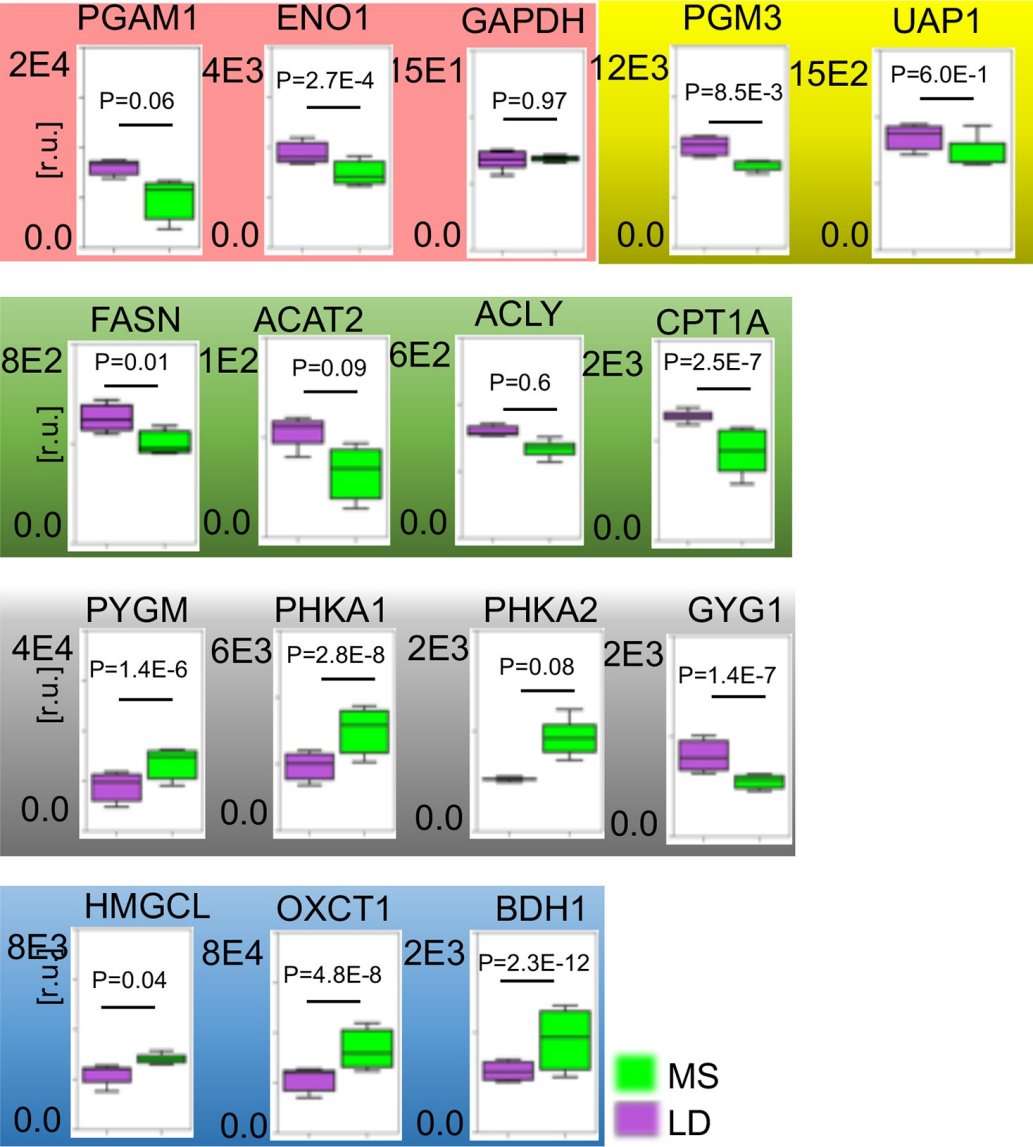
Myocardial mRNA response to MetS. RNA-seq is applied to evaluate relative changes in mRNA levels of enzymes in myocardial MetS and LD tissue. Plots show mRNA levels in MetS (green barrels) versus LD (purple) of four individual MetS swine and four LD swine; PGAM1(p = 6.2x10^-2^), ENO1 (p = 2.7x10^-4^), GAPDH (p = 0.97); PGM3 (p = 8.5x10^-3^), UAP1 (p = 6x10^-1^), FASN (p = 0.01); ACAT2 (p = 0.09), ACLY (p = 0.6), CPT1A (p = 2.5x10^-7^), HMGCL (p = 0.04), OXCT1 (p = 4.8x10^-8^), BDH1 (2.4x10^-12^), PYGM (p = 1.4x10^-6^), PHKA1 (p = 2.8x10^-8^), PHKA2 (p = 0.08) and GYG1 (p = 1.4x10^-7^). Data are presented as means ±SD (normalized counts LD = 4, MetS = 4; p<0.05 were considered statistically significant; p_adj_ reported in supporting S2 Table). The mRNA identity is shown above the plots. mRNAs for enzymes with functions in the glycolysis pathway are highlighted in pink; hexosamine biosynthetic pathway in yellow; green—fatty acids metabolism; glycogen breakdown, metabolism, and synthesis in gray; mitochondrial ketone body formation, transport, and utilization in blue.

As most of the differentially present metabolites are involved in glycolysis, HBP, fatty acids metabolism, and ketogenesis we next compared the mRNA expression levels of enzymes that are involved in these pathways (Fig 4). The RNA-seq data derived from sequencing left ventricular myocardial mRNA libraries obtained from 4 swine of each group show a decrease in the glycolysis related phosphoglycerate mutase in MetS (PGAM1) mRNA (p = 6.2x10^-2^) and ENO1 (p = 2.7x10^-4^) in MetS. The expression of GAPDH (p = 0.97) however did not show diet dependent alteration in the myocardium and add value to the other gene expression data. The PGM3 expression (p = 8.5x10^-3^) together with UAP1 (p = 6x10^-1^) from the HBP pathway that control the synthesis of the substrate for N-linked and O-linked protein glycosylation are both significantly less expressed in MetS vs. LD. The analyses also consistently show less mRNA in MetS for enzymes that are involved in fatty acid synthesis and beta-oxidation: fatty acids synthase (FASN, p = 0.01) and the essential for fatty acid oxidation carnitine palmitoyltransferase 1A (CPT1A, p = 2.5x10^-7^). Acetyl-CoA acetyltransferase 2 (ACAT2, p = 0.09) and ATP citrate lyase (ACLY, p = 0.6) show tendency for decrease (Supporting S2 Table). While glycolysis and fatty acid metabolism-related enzyme expression is down regulated in MetS, the mitochondrial HMGLC (p = 0.04), OXCT1 (p = 4.8x10^-8^), and BDH1 (p = 2.4x10^-12^) are significantly upregulated. Similarly, the expression of PYGM (p = 1.4x10^-6^), PHKA1 (p = 2.8x10^-8^), and PHKA2 (p = 0.08) that are in control of the glycogen depletion is significantly higher in MetS vs. LD. The glycogenin GYG1 (p = 1.4x10^-7^) mRNA that is essential for the synthesis of glycogen, however, is significantly less expressed in MetS vs LD.

Taken together, these data support the perception of activated ketone body synthesis and diminished glycolysis, fatty acids and glycogenolysis in MetS due to altered gene expression of traditional enzymes. Gene expression data suggests for diminished HBP enzymatic activity that contributes to the low availability of UDP-GlcNAc, the protein O- GlcNAcylation substrate in MetS.

### Global protein O-GlcNAcylation response to MetS in the pig heart

**Fig 5:**
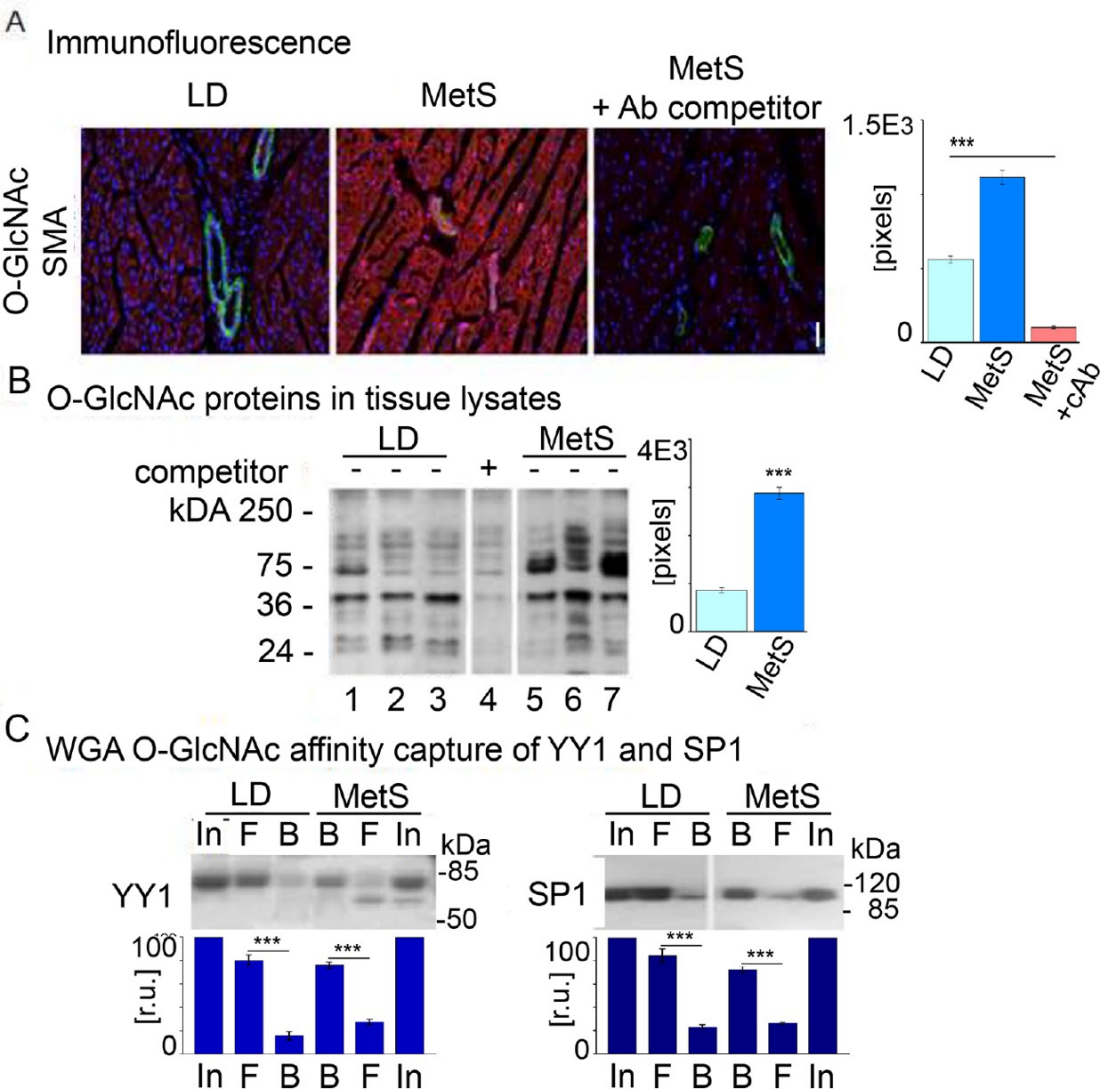
Cardiac protein O-GlcNAcylation in MetS and LD conditions. LD and MetS cardiac tissues differ in content of O-GlcNAcylated proteins. (A) Paraffin embedded tissue sections were stained with anti-O-GlcNAcylated antibody (red) and smooth muscle alpha actin (green). DNA was stained with DAPI (blue). The identity of the tissue sections (LD–lean diet control, MetS–animals fed a high fat diet, and MetS+Ab competitor- sections of MetS swine after antibody competitor N-acetylglucosamine administration) is shown at the top. The bars show the average value of representative independent tissue sections from MetS and LD (n = 6 per group) and MetS that received antibody competitor (cAb). (B) Western blot with tissue lysates (50 μg protein/lane) from three representative LD pigs (lanes 1, 2, 3) and three MetS pigs (lanes 5, 6, 7); lane 4 shows lysate after administration of 5 μg N- acetylglucosamine as a competitor. The bars on right present the average relative values of specific antibody signals. Data are represented as means ±SD; ***p<0.001. Ponceau staining is shown in S2 Fig in the Supporting. (C) WGA affinity capture of O-GlcNAcylated proteins in MetS and LD tissue lysates. Captured proteins were eluted and analyzed by Western blot for presence of YY1 and Sp1: input (IN); unbound flow through (F); bound (B). The YY1 and Sp1 position is indicated on the left of the panels. The relative amount of YY1 and Sp1 in the fractions is shown as an average (pixels) in the diagrams below the panels. The amount of YY1 and Sp1 in the input was used as a base (100%) for comparison. The immunohistochemistry data for O-glycosylation are presented as average mean intensity of the specific immunological reaction (in pixels)/40XHPF ± SD. The calculations were conducted in NIH Image 1.31. Data are representative of three independent experiments with lysates from three individual MetS and respectively LD swine. Data are represented as means ± SD; ***p<0.001.

Reduced availability together with the diminished protein O- GlcNAcylation substrate UDP-GlcNAc in MetS is expected to suppress cardiac protein O- GlcNAcylation. We applied three different approaches to examine protein O-GlcNAcylation in MetS versus LD pigs. Immunofluorescence experiments with O-GlcNAc-specific antibody and left ventricle (LV) cryosections (Fig 5A) show that a significant number of cells (>70%, p<0.001, n = 4) stain positively with the antibody in the MetS sections. A nearly 4-fold decrease of cells that stain positively (16%, p<0.001, n = 4) is observed in LD sections. Anti-O-GlcNAc antibody is specific to its ligand as demonstrated by the significant reduction in staining with its pretreatment with N-acetylglucosamine (<5%, p<0.001, n = 4). In the second assay, we performed Western blot experiments with MetS and LD pig lysates and an anti-O-GlcNAc antibody (Fig 5B). The presence of O-GlcNAcylated proteins was quantified by measuring the optical (pixel) density of the positive immunostaining reactions in Western blots with equal amounts (50 μg/well) of protein. N-Acetylglucosamine pretreatment of the O-GlcNAc antibody served as the negative control, and its densities were subtracted from those of the antibody reactions as background (Fig 5B, lane 4). We used Ponceau S staining profile of the transferred membrane proteins as an internal control for equal protein loading (S1 Fig in Supporting information). Again, we observed a significantly higher O-GlcNAcylated protein content in MetS lysates while LD lysates exhibited reduced reactivity to the O-GlcNAc antibody. O-GlcNAcylated protein measurements are 3.54-fold higher (p<0.001, n = 4) in MetS compared to LD. These results are consistent throughout independent experiments with four MetS and four LD tissue lysates. When wheat germ agglutinin (WGA) affinity captured O-GlcNAcylated proteins are stained via Western blot with YY1- and Sp1-specific antibodies (Fig 5C), lysates from MetS swine exhibit higher levels of O-GlcNAcylated YY1 and Sp1 variants compared to those of LD swine (Fig 5C, lanes B). Despite the significantly reduced availability of G6P, F6P, GlcNAc-1p, and UDP-Glc which are key metabolites in glycolysis and HBP, the results show a robust activation of cardiac pan- protein O-GlcNAcylation in MetS. Importantly, the MetS conditions induced by high-fat-diet appear to be associated with these modifications of transcription factors YY1 and Sp1, which could trigger metabolic and inflammatory changes in gene expression [12–15]. As the increased protein O-GlcNAcylation cannot be explained with the diminished G6P, F6P, and substrate UDP-GlcNAc abundance, it is likely that the O-linked N- acetylglucosaminyltransfrerase (OGT) and OGA availability balance could elucidate the protein O-GlcNAcylation discrepancy in high-fat-diet pigs.

### MetS and LD tissues significantly differ in their content of OGA

**Fig 6:**
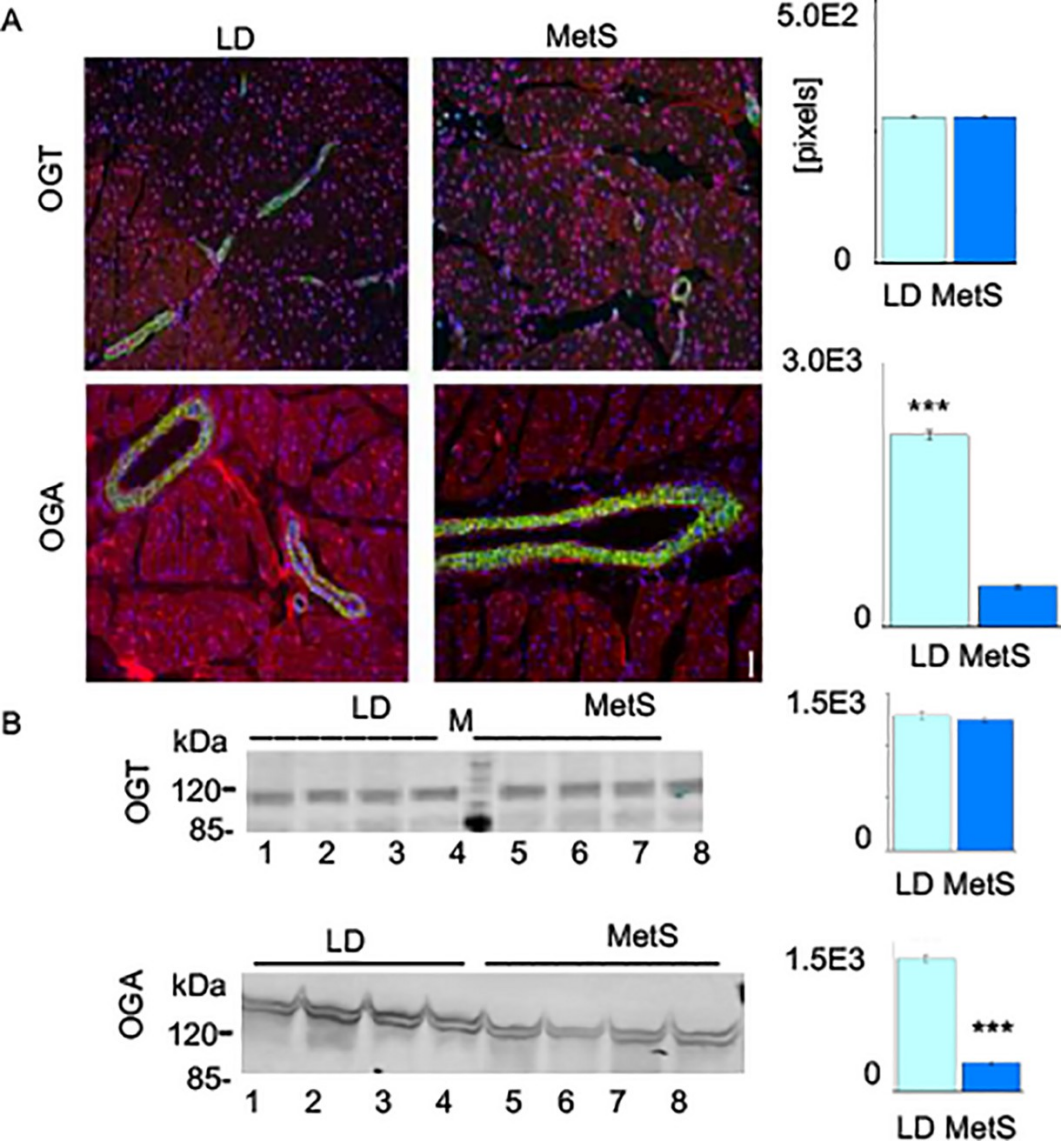
OGT and OGA content in LD and MetS cardiac tissues. (A) Immunofluorescence showing differences in OGA content (red) in LD and MetS tissue while their OGT content (red) is nearly identical: green shows smooth muscle alpha actin antibody and blue shows the nuclear DNA. The intensity of the red staining in a surface with equal number of nuclei was measured with NIH ImageJ 1.31 and the bar diagram at the right presents the average OGT and OGA staining value of tissue sections from MetS pigs presented as relative pixels (n = 4, ***p<0.001). (B) Western blot showing amount of OGT and OGA in tissue lysates (50 **μ**g protein/lane) from four representative LD pigs (line 1, 2, 3, 4) and four MetS pigs (lines 5, 6, 7, 8). The bars on right present pixel density of OGA and OGT in MetS lysates vs. LD and determined by ImageJ 1.31 and are shown as mean ± SD (n = 4, ***p<0.001). The p values were calculated by Studentʼs t-test. See also Supporting information S2 Fig.

As alterations in the availability of OGT and OGA may rationalize the enhanced protein O- GlcNAcylation in MetS pigs, we conducted immunofluorescence staining experiments on MetS and LD tissue sections using antibodies specific to these two enzymes (Fig 6). The results demonstrate that similar levels of immunologically reactive OGT are detectable in both MetS and LD tissue sections (Fig 6A). Contrary to this finding, protein levels for its partner OGA are more than 4-fold lower in MetS (n = 4, p<0.001) than in LD. To further examine this discrepancy, Western blots with MetS and LD tissue lysates were performed (Fig 6B) and showed greater than 5-fold decrease in the content of immunoreactive OGA in MetS tissue (n = 4, p<0.001).

Taken together, these outcomes allude to MetS- specific balance between OGT and OGA that favors a higher level of protein O-GlcNAcylation despite the substantially decreased availability of glucose metabolites. These data point to a myocardial obligation in MetS to O-GlcNAcylate cardiac proteins by altering the balance between OGT and OGA.

## Discussion

Genetics and signaling pathway analysis have been successfully leveraged in the study of MetS to implicate pleotropic factors that underlie its development. Though this interrogation has involved many processes in MetS, including aberrant gene expression, mitochondrial function, ion channel regulation, hemodynamics, and electrophysiology, the complexity of the implicated symptoms is such that essential mechanisms that drive the development of MetS remain to be characterized. Our experimental and NMF computational strategy of metabolites from a swine model of MetS represents an important step in further elucidating the effects of MetS on the myocardium.

Though mass spectrometry is a robust tool that enables simultaneous quantification of many metabolites, statistical analysis of the obtained data is often challenging as the number of variables is usually more than the number of replicates. Our application of NMF to 280 hydrophilic metabolites from the swine myocardium is novel in comparison to many commonly employed multivariate statistics and pattern recognition approaches such as random forest, false discovery rates, and receiver operator character analysis. These techniques represent supervised analysis that can only detect alternations in signaling of known pathways. The NMF approach is agnostic to pathways and enables the characterization of previously undescribed metabolite networks. In applying this approach to our myocardium obtained from both MetS and LD swine we are able to catalog differences in metabolite profiles between these groups and describe the energy status of the MetS myocardium.

Specifically, our NMF clustering of polar metabolites reveals that in MetS (P4) there is an unexpected decline in metabolites related to fatty acid oxidation and fatty acid synthesis, as well as concurrent decline in glycolysis and glycogenolysis as evidenced by reduced acetyl- CoA, malonyl-CoA, propionyl-CoA, ATP, pyruvate, lactate, and glycogen deposition. Currently, the etiology of repressed myocardial fatty acid metabolism in MetS is not well understood. In the conditions of hyperglycemia, the unbalanced malonyl-CoA could have an effect on both fatty acid synthesis and mitochondrial fatty acid oxidation. The elevated circulating triglycerides and fatty acids in MetS may overwhelm mitochondrial capacity for fatty acid oxidation [16], causing subsequent dysfunction, though further investigation is warranted.

Metabolite profiles in MetS show significantly diminished G6P, F6P, and FBP1– key intermediates in glycolysis. Furthermore, G3P generated from the ATP producing steps in glycolysis and the final glycolysis pathway product, pyruvate is reduced in MetS.

Finally, as pyruvate and the pyruvate derived lactate in MetS are both low, compensatory glycogenesis in MetS is unlikely to proceed [17].

Importantly, hyperglycemia in MetS further diminishes glucose flux through glycolysis, indirectly shrinking another energy source for the heart, glycogen. In the conditions of increased free fatty acid availability in MetS we registered statistically significant decrease in G1P levels together with the significantly diminished glycogen availability.

Despite diminished availability of HBP intermediates including UDP-GlcNAc, the substrate for protein O-GlcNAcylation, we observed more O-GlcNAcylated proteins in MetS. The pig myocardium may respond to the high fat diet induced MetS by altering protein O-GlcNAcylation as previously observed in a rat model of the disease [18]. The ubiquitous transcription factors YY1 and Sp1 are mostly O-GlcNAcylated as well. YY1 regulates the expression of several mitochondrial genes [14, 15] and its O- GlcNAcylation could alter its functions as a transcription regulator in a gene promoter specific way by altering interactions with other transcriptional regulators [12, 15]. Many analyzed mitochondrial promoters show functional GC-boxes, which bind Sp1. O-GlcNAcylation was shown to down-regulate Sp1 activity [13, 14]. Exactly how the O-GlcNAcylated YY1 and Sp1 variants participate in the regulation of enzymes that are essential to glycolysis and the MetS phenotype remains to be established.

The higher abundance of O-GlcNAcylated proteins is consistent with diminished availability of OGA in MetS, the enzyme which removes O-GlcNAc moieties from target proteins. Though myocardial OGA is dysregulated in MetS, its co-regulation with the OGT-OGA ratio may improve myocardial energy balance and be of therapeutic relevance.

In the context of diminished glucose and fatty acids oxidation intermediates in MetS we register higher presence of the ketone metabolites acetoacetate and 3-hydroxybutirate. The reason for this is unknown but could reflect higher ketone oxidation rates related to activated HMG-CoA lyase expression and increased ketone body delivery through the blood and increased reliance on ketone bodies as a fuel in MetS. Consistent with this notion, a recent study demonstrated that the mouse myocardium increasingly relies on ketone bodies as a fuel during heart failure and reduced capacity for oxidizing fatty acids [19]. The myocardial specificity of metabolic alterations, however, should be verified in other tissues as well as in the blood stream.

A general question arises as to whether the low metabolites abundance reflects a higher rate of consumption in MetS. The RNA-seq data supports the perception of diminished pathways activity due to altered gene expression of pathways enzymes rather than high consumption rates. PGAM1 that controls step eight of glycolysis is downregulated in MetS and could partly contribute to the decrease availability of intermediates and the final glycolysis product pyruvate. The MetS effect on PGAM1 mRNA expression in the myocardium is supported by the recently reported decreased glycolysis and pentose phosphate pathway flux in response to transcriptional inhibition of PGAM1 [20]. The cytoplasmic ENO1 (p = 0.03) that catalyzes the dehydration of PGA to phosphoenolpyruvate in the catabolic direction of the glycolytic pathway is down regulated in MetS as well.

The fatty acid oxidation and fatty acids synthesis related CPT1A (p = 0.01), ACAT2 (p = 0.01), FASN (p = 0.01), and ACLY (p = 0.01) are all transcriptionally downregulated in MetS that would contribute the low acetyl-CoA availability. The glycogen synthesis-glycogen degradation balance in the myocardium is clearly disturbed in MetS. The higher expression of PYGM, PHKA 1 and 2 together mediate the final stages of glycogen degradation. The expression of the essential in the glycogen biosynthesis glycogenin1 (GYG1) however, is transcriptionally downregulated and fails to compensate the degradation resulting in glycogen depletion in MetS. Advancing understanding of glycogen dynamics in the heart is an important priority not only with relevance to MetS but also to cardiac metabolic stress and pathology [21]. The expression of the essential in the ketone body formation enzyme HMG-CoA lyase (HMGCL) (p = 0.01) and its myocardial isoform HMGCLL1 (p = 0.01) however, are highly expressed in the MetS. The mitochondrial matrix enzyme OXCT1 that plays a central role in in the ketone body catabolism and the mitochondrial BDH1 that catalyzes the interconversion of acetoacetate and 3- hydroxybutyrate, the two major ketone bodies are both highly expressed in MetS. We observed that the monocarboxylate transporter SLC16A3 that catalyzes the rapid transport of carboxylates including acetoacetate and 3-hydroxybutyrate across the plasma membrane is highly abundant in MetS [22]. How much and how exactly the metabolites utilization rate contributes to the process remains to be determined in a different experimental setting. Moreover recently published observations supported the role of ketone bodies as an energy source in diabetic patients as well [23].

## Conclusion

Overall, these data point out to a transcriptionally controlled fuel gauge in the MetS heart that enzymatically turns down glucose and fatty acids oxidation and activates nutritional ketolysis.

Although the hexosamine biosynthetic pathway (HBP) activity and the availability of the UDP- GlcNAc substrate in the MetS myocardium is low the level of O-GlcNacylated proteins is high as the O-GlcNAcase (OGA) expression is significantly diminished. Overall, our data and analysis provide a picture of the MetS identity in the myocardium and do represent a major step forward uncovering targets for intervention and control of the myocardial disease processes in MetS. Together, this study represents a significant step towards characterizing the metabolic state of the heart in MetS and provides potential avenues for therapeutic advance.

## Materials and methods

### Animal model

Eight weeks old male intact Yorkshire swine (n = 6) (Sinclair Research, Columbia, MO) were fed an obesogenic 2248 kcal/d (hypercholesterolemic) diet daily: 500 grams of feed composed of 4% cholesterol, 17.2% coconut oil, 2.3% corn oil, 1.5% sodium cholate and 75% regular chow (Sinclair Research, Columbia, MO) for 12 weeks and was used to model MetS. Control LD animals (n = 6) were given regular chow (500 g/d; 1824 kcal/d). After 12 weeks, high fat diet model induce obesity, hyperlipidemia, high blood pressure, insulin resistance, and glucose intolerance which are components of MetS, swine were anesthetized by exsanguination following removal of the heart while under deep isoflurane anesthesia and physiologic measurements were taken. The myocardial tissue samples from all animals were taken from identical myocardial left ventricular territories (LV) and were rapidly frozen in liquid nitrogen. All experiments were approved by the Institutional Animal Care and Use Committee of the Rhode Island Hospital. Animals were cared for in compliance with the “Principles of Laboratory Animal Care” formulated by the National Society for Medical Research and the “Guide for the Care and Use of Laboratory Animals” (NIH publication number 5377–3, 1996) [24].

### Serological analyses

Blood samples were drawn from the jugular vein prior to euthanasia and tissue harvest for insulin measurement. The chemistry laboratory at the Rhode Island Hospital, Providence, RI, analyzed the serum samples.

### Immunofluorescence, Western blotting, WGA affinity chromatography

Frozen sections (12 μm in thickness) or formalin fixed tissue sections of myocardium from the non-ischemic territories were stained with anti- MGEA5 (OGA) antibody [EPR7154 (B)], (Abcam); anti-OGT (ARP49154_P050- FITC, AVIVA); Anti-O-Linked N-Acetylglucosamine Antibody, clone RL2 (SigmaAldrich); α- Smooth Muscle actin—FITC antibody (Vector); anti- YY1 antibody—ChIP Grade (ab38422) (ABcam); Anti-SP1 antibody—ChIP Grade (ab13370) (ABcam). WGA affinity chromatography was conducted as in [12, 25]. The equal loading and transfer are monitored by staining the membranes after transfer with Ponceau S. The protein concentration is measured with the BCA Assay kit (Pierce). The protein expression analyses for OGT and OGA show the 4 animals that has been analyzed for gene expression. In Fig 5B and 5C the western blot was conducted using one 10 wells SDS gel: 3 LD lysates from randomly selected pigs–lanes 1, 2, 3; four MetS lysates (lines 4, 5, 6, 7); two border lanes with pre-stained markers. Panel 4 (line 4) is an antibody specificity control. It is loaded with MetS lysate #7 and incubated with the antibody and N-acetylglucosamine as a competitor.

### RNA-seq

Total RNA was extracted from fresh frozen left ventricular myocardial tissue QIAshredder (Qiagen) in combination with RNeasy Mini Kit (Qiagen), with on-column DNase digestion according to the manufacturer's protocol. RNA integrity was assessed using an RNA 6000 Nano Kit (Agilent Technologies) on a Bioanalyzer (Agilent Technologies). All 8 samples (4 MetS and 4 LD) submitted for sequencing (eight independent RNA isolations and samples) had RNA integrity ≥ 9.8. mRNA libraries were sequenced with 2x50bp paired-end reads on an Illumina HiSeq 2500 in high-output mode to an average range dept of 86x10^6^ paired-end reads/sample, with a range of 43x10^6^-139x10^6^ at GENEWIZ (South Plainfield, NJ). The reads were mapped to the porcine reference genome (USMARCv1.0) using the STAR aligner [26], and quantified read counts for all genes annotated (USMARCv1.0) with HTSeq- count, version 0.5.3p9 [27]. Differential gene expression analysis was performed at GENEWIZ with the Bioconductor package DESeq. The selection of animal sample for RNA-seq was random.

### Two-dimensional thin layer chromatography (TLC)

TLC was conducted with the extracted polar metabolites on silica gel G plates with fluorescent indicator at 254 nm (Sigma-Aldrich). The first dimension separation was conducted in an acid solvent system (ethanol– 5N NH4OH- H2O) ratio 200:9:40; second dimension in a basic solvent system (insolently formate-formic acid-water) ratio 110:20:5. Spots are identified in UV light on the migration of the standard molecule alone.

### Periodic Acid-Schiff (PAS), Lipid Oil Red O Staining, Picrosirius Red Staining

The Periodic Acid-Schiff (PAS) staining was performed with the PAS staining system from Sigma-Aldrich (procedure 395). In the cardiac tissue, the staining results mainly from reaction with glycogen although other carbohydrate with 6 macromolecules could react as well. Lipid (Oil Red O) Staining Kit (Bio Vision, Catalog # K580-24) was used to stain for lipids accumulation. Picrosirius Red Staining was conducted with kit from Polysciences, Inc. Four slides per animal have been used to quantify the data; four images from random area per slide were captured and the mean positive staining was determined using Image J software.

### Polar metabolites LC/MS-MS

Polar metabolites were extracted from 100 mg flash-frozen tissue with 1ml of ice-cold 80% (v/v) methanol and 0.6 ml acetonitrile and analyzed using a 5500 QTRAP hybrid triple quadrupole mass spectrometer (AB/SCIEX) coupled to a Prominence UFLC HPLC system (Shimadzu) with SRM as in [8]. Peak areas from the total ion current for each metabolite SRM transition were integrated using MultiQuant v2.0 software (AB/SCIEX). LC/MS-MS was conducted for the individuals pig samples (12 pigs in 12 independent runs). MetaboAnalyst 4.0 was used to identify known pathways participation.

### Nonnegative matrix factorization (NMF)

Nonnegative matrix factorization (NMF) was applied as shown in [10] to search for metabolic signatures in the LC/MS-MS data set of 280 metabolites in the analyzed Yorkshire pigs (MetS n = 6, LD n = 6) total 12 pigs. Hierarchical clustering was performed as in [26, 28]. All simulations were run on Linux clusters at the Los Alamos National Laboratory.

### Statistical analysis

Data analysis was performed using Microsoft Excel and Graphpad Prism7 software. For statistical analysis, Student’s t test (GraphPad Software, Inc, San Diego, CA) was used to compare differences between 2 datasets. Data are presented as means ± SD. In all cases, p < 0.05 was considered to be a statistically significant difference. For transcriptome data analysis, p value was used to compare differences between 2 datasets (p value<0.05; supporting S2 Table). Data are presented as means ± SD. Immunohistochemistry for O-glycosylation, OGA, OGT, are presented as average mean intensity (in pixels)/40XHPF +/− SD or as a fold change vs. LD. Six random fields were analyzed for each pig. Western blot data are presented as fold MetS change (pixels, +/− standard error of the mean, SD) vs. LD.

## Data access

The RNA-Seq data are available under GEO accession number PRJNA544355. The NMF predicted metabolites with higher probability in LD vs MetS (P4 = 0) are shown in the Supporting information (S1 Table). The 280 polar metabolites probability distribution in MetS and LD are shown in the supporting information (S2 Table).

## Supporting information

S1 Fig Cardiac protein O-GlcNAcylation in MetS and LD conditions (related to Fig 5B and 5C). The Ponceau S stain confirmed equal loading of input proteins on each of the Western blot lines with tissue extracts from LD pigs (lane 1, 2, 3) and MetS pigs (lane 5, 6, 7); lane 4 shows lysate from line 7 after incubation with antibody mix containing 5 μg N- acetylglucosamine as a competitor.
(PDF)

S2 Fig Ponceau S stain and Western blot with protein specific antibodies against OGT, OGA and GAPDH and LD and MetS cardiac tissues (related to Fig 6B). The Ponceau S stain confirmed equal loading of input proteins on each of the Western blot lines with tissue extracts from LD pigs and MetS pigs.
(PDF)

S1 Table Related to Fig 2A. Weights of the metabolites in the P2 (LD) and P4 (MetS) NMF signatures. All metabolites are quantitatively presented in the individual signatures with a signature specific relative weight. The relative weight of all metabolites in a signature is equal 1. The Supplemental S1 Table provides the weight distribution of all metabolites in signature P2 and P4. Both signatures are directly related to the main subject of the manuscript.
(PDF)

S2 Table Related to Fig 4. RNA-Seq reads over multiple runs. Plots show entire transcriptome RNA-Seq reads for LD (Lanes 1–4) across 4 technical replicates and for MetS across 4 replicates (lanes 5–8).
(PDF)

## Abbreviations

MetS: Metabolic Syndrom
LD: Lean Diet control
G6P: Glucose-6-phosphate
F6P: Fructose 6-phosphate
AcCoA: Acetyl coenzyme A
Propionyl CoA: Propionyl Coenzyme A
Malonyl CoA: Malonyl Coenzyme A
FBP1: fructose-1,6-bisphosphatase
G3P: Glyceraldehyde 3-phosphate
ATP: Adenosine Tri-Phosphate
G1P: Glucose 1 Phosphate
UDP-GlcNAc: Uridine diphosphate *N*-acetylglucosamine
GlcNAc-1P: N-Ac-Glucosamine-1-phosphate
PGAM1: Phosphoglycerate Mutase 1
ENO1: Enolase 1
GAPDH: Glyceraldehyde 3-phosphate dehydrogenase
CPT1A: Carnitine Palmitoyltransferase 1A
ACAT2: Acetyl-CoA Acetyltransferase 2
FASN: Fatty Acid Synthase
ACLY: ATP Citrate Lyase
OXCT1: 3-Oxoacid CoA-Transferase 1
BDH1: 3-hydroxymethyl-3-methylglutaryl-CoA lyase
HMGCL: 3-hydroxy-3-methylglutaryl-CoA lyase
PYGM: Glycogen Phosphorylase
PHKA1: Phosphorylase Kinase Regulatory Subunit Alpha 1
PHKA2: Phosphorylase Kinase Regulatory Subunit Alpha 2
UAP1: UDP-N-Acetylglucosamine Pyrophosphorylase 1
HBP: hexosamine biosynthetic pathway
PPP: pentose phosphate pathway
NMF: Nonnegative matrix factorization
MetS: Metabolic syndrome
OGA: O-GlcNAcase
LV: left ventricle
WGA: wheat germ agglutinin
OGT: N-acetylglucosaminyltransfrerase
GYG1: glycogen biosynthesis glycogenin1
PAS: Periodic Acid-Schiff
