# Supplementary figures and images for: Robust effect of metabolic syndrome on major metabolic pathways in the myocardium
Source: PLoS One. 2019 Dec 2;14(12):e0225857. doi: 10.1371/journal.pone.0225857 (PMC6886832; doi:10.1371/journal.pone.0225857)

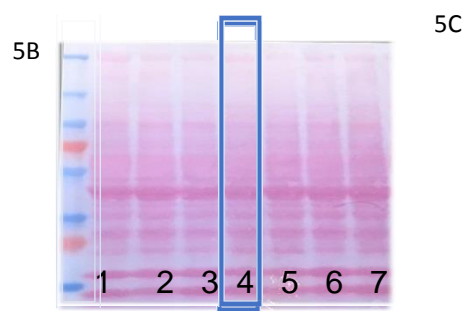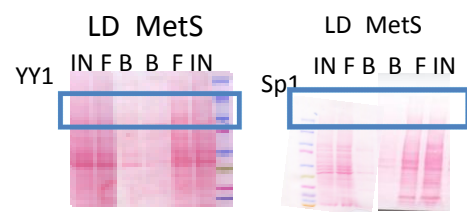

Supplement: S1 Fig — The Ponceau S stain confirmed equal loading of input proteins on each of the Western blot lines with tissue extracts from LD pigs (lane 1, 2, 3) and MetS pigs (lane 5, 6, 7); lane 4 shows lysate from line 7 after incubation with antibody mix containing 5 μg N- acetylglucosamine as a competitor. (PDF) [file pone.0225857.s001.pdf]

LD M MetS

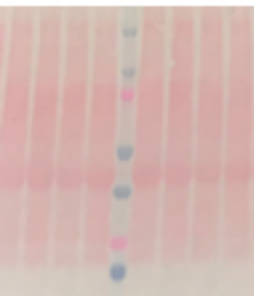

OGT

LD MetS

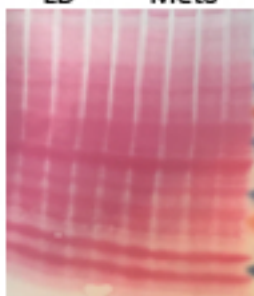

OGA

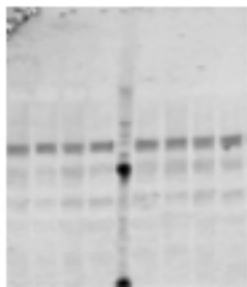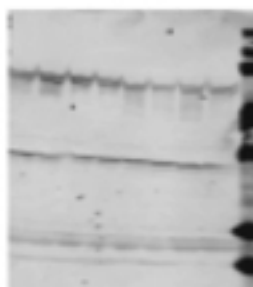

-GAPDH

Supplement: S2 Fig — The Ponceau S stain confirmed equal loading of input proteins on each of the Western blot lines with tissue extracts from LD pigs and MetS pigs. (PDF) [file pone.0225857.s002.pdf]
