# Supplementary material for: Robust effect of metabolic syndrome on major metabolic pathways in the myocardium
Source: PLoS One. 2019 Dec 2;14(12):e0225857. doi: 10.1371/journal.pone.0225857 (PMC6886832; doi:10.1371/journal.pone.0225857)
Supplement: S1 Table — Weights of the metabolites in the P2 (LD) and P4 (MetS) NMF signatures. All metabolites are quantitatively presented in the individual signatures with a signature specific relative weight. The relative weight of all metabolites in a signature is equal 1. The Supplemental S1 Table provides the weight distribution of all metabolites in signature P2 and P4. Both signatures are directly related to the main subject of the manuscript. (PDF) [file pone.0225857.s003.pdf]

signature name	P2	P4
1,3-diphopshateglycerate	0	0.000418
1-Methyladenosine	0.000064	0.000086
1-Methyl-Histidine	0.003226	0.003603
2,3-dihydroxybenzoic acid	0.000001	0.000007
2,3-Diphosphoglyceric acid	0	0.000468
2-Aminooctanoic acid	0.000055	0.000374
2-dehydro-D-gluconate	0.000007	0.000028
2-deoxyglucose-6-phosphate	0.00138	0.001589
2-Hydroxy-2-methylbutanedioic acid	0.002339	0.000263
2-hydroxygluterate	0.007995	0.000898
2-Isopropylmalic acid	0.000029	0.000041
2-ketohaxanoic acid	0.000181	0.000059
2-keto-isovalerate	0.001661	0.000817
2-oxo-4-methylthiobutanoate	0.000018	0
2-oxobutanoate	0.000269	0.00013
3-hydroxy-3-methylglutaryl-CoA-nega	0.000006	0.000003
3-hydroxybuterate	0.00013	0.000147
3-hydroxybutyryl-CoA	0.000012	0.000013
3-methylphenylacetic acid	0.000007	0.000007
3-phosphoglycerate	0.000364	0.000104
3-phospho-serine	0.000007	0.000003
3-S-methylthiopropionate	0.000002	0.000002
4-aminobutyrate	0.000024	0.000022
4-phosphopantothenate	0.000024	0.000021
4-Pyridoxic acid	0.000091	0.000025
5-methoxytryptophan	0.000014	0.000011
5-methyl-THF	0.000015	0.000009
5-phosphoribosyl-1-pyrophosphate	0.000029	0.000029
6-phospho-D-gluconate	0.000064	0.000018
7,8-dihydrofolate	0.000001	0
7-methylguanosine	0.000058	0.000089
acadesine	0.000002	0.000002
acetoacetate	0.000255	0.000312
acetoacetyl-CoA	0	0.000002
Acetylcarnitine DL	0.035618	0.034604
acetyl-CoA	0.000509	0
Acetyllysine	0.007446	0.020745
acetylphosphate	0.000408	0.000469
aconitate	0.00008	0.000341
adenine	0.002409	0.002524
adenosine	0.000054	0.000077
adenosine 5-phosphosulfate	0.000062	0.000185

Adenylosuccinate	0.000007	0.000001
ADP-D-glucose	0	0.000029
ADP	0.006847	0.017663
a-ketoglutarate	0.00504	0.004938
alanine	0.015739	0.014656
allantoate	0.000175	0.000016
allantoin	0.000161	0.000451
Aminoadipic acid	0.000013	0.000156
aminoimidazole carboxamide ribonucleotide	0.000004	0.000003
AMP	0.000964	0.030458
anthranilate	0.000218	0.000275
arginine	0.005167	0.009418
arginosuccinic acid	0.000167	0.000192
Ascorbic acid	0.000898	0.000438
asparagine	0.000026	0.000351
aspartate	0.000529	0.00087
ATP	0.036561	0.026251
Atrolactic acid	0.000004	0.000003
betaine	0.034744	0.026748
betaine aldehyde	0.00027	0.000383
biotin	0.000002	0.00002
butyryl-CoA	0.000031	0.000007
Carbamoyl phosphate	0.008963	0.010002
carnitine	0.02204	0.028599
CDP-choline	0.000057	0.000107
CDP-ethanolamine	0.00007	0.000031
CDP-nega	0.000008	0.000049
Cellobiose	0.00028	0.000091
cholesterol	0.00007	0.000101
cholesteryl sulfate	0.000094	0.000233
Cholic acid	0.000002	0.000001
choline	0.000316	0.000648
Citraconic acid	0.000915	0.001333
citrate	0.003995	0.009758
citrate-isocitrate	0.009562	0.021154
citrulline	0.003379	0.003151
CMP	0.000465	0.000232
coenzyme A	0.000009	0.000033
creatine	0.078854	0.09119
Creatinine	0.003943	0.003316
CTP	0.000072	0.000213
cyclic bis(3->5) dimeric GMP	0.000001	0
cyclic-AMP	0.00035	0.000192

cystathionine	0.000001	0
cysteine	0.000003	0.000003
Cystine	0.000016	0.00003
cytidine	0.004529	0.001459
cytosine	0.002709	0.000884
dAMP	0.000117	0.000094
dATP	0.000019	0.000004
dCDP-	0.000007	0.000009
dCMP	0.000008	0.000002
dCTP	0.000005	0.000023
dehydroascorbic acid	0.000002	0
deoxyadenosine	0.00001	0.000003
deoxyguanosine	0.00001	0.000009
deoxyinosine	0.000158	0.000204
deoxyribose-phosphate	0.000068	0.000092
deoxyuridine	0.000073	0.000044
dephospho-CoA	0	0.000275
D-erythrose-4-phosphate	0.000163	0.00006
dGDP	0.007212	0.017475
D-glucarate	0.000048	0.00006
D-gluconate	0.000038	0.000242
D-glucono-?-lactone-6-phosphate	0.000021	0.000006
D-glucosamine-1-phosphate	0	0.000002
D-glucosamine-6-phosphate	0.000036	0.000009
D-glyceraldehdye-3-phosphate	0.00012	0.000108
dGMP	0.000001	0.000003
dGTP	0.037415	0.026762
dihydroorotate	0.000114	0.000234
dihydroxy-acetone-phosphate	0.000505	0.000341
dimethylglycine	0.000242	0.000082
DL-Pipecolic acid	0.012077	0.015218
D-sedoheptulose-1-7-phosphate	0	0.000189
dTDP	0.000006	0.000011
dTMP	0.000001	0.000005
dTTP	0.000031	0.000012
dUMP	0.000001	0.000003
dUTP	0.000013	0.000007
ethanolamine	0.000073	0.00014
FAD	0.001406	0.000597
Flavone	0.000002	0.000002
FMN	0.000011	0.000001
folate	0	0.000004
fructose-1,6-bisphosphate	0.001644	0.00078

fructose-6-phosphate	0.00079	0.000177
fumarate	0.001717	0.000861
GDP	0.000073	0.000352
Geranyl-PP	0	0.000227
glucono-?-lactone	0.000003	0.000011
glucosamine	0.000033	0.000206
glucose-1-phosphate	0	0.000211
glucose-6-phosphate	0.001008	0.000324
glutamate	0.012461	0.016798
glutamine	0.020552	0.027212
glutathione	0.000145	0.000001
glutathione disulfide	0.01414	0.007843
glutathione	0.000278	0.000165
glycerate	0.000272	0.000415
Glycerophosphocholine	0.028845	0.043559
glycine	0.000002	0.000003
glycolate	0.000029	0.000033
glyoxylate	0.000037	0.000021
GMP	0.000021	0.000235
GTP	0.001045	0.001069
Guanidoacetic acid	0.000435	0.000168
guanine	0.000356	0.000166
guanosine	0.000117	0.000053
guanosine 5-diphosphate,3-diphosphate	0	0.000045
hexose-phosphate	0.006456	0.002689
histidine	0.004845	0.004883
histidinol	0.000003	0.00001
homocysteic acid	0.000294	0
homocysteine	0.000002	0.000005
homoserine	0.000002	0.000003
Hydroxyisocaproic acid	0.000031	0.000031
Hydroxyphenylacetic acid	0.000014	0
hydroxyphenylpyruvate	0.000014	0.000009
hydroxyproline	0.000444	0.000424
hypoxanthine	0.013835	0.006986
IDP	0.000921	0.002647
Imidazoleacetic acid	0.000021	0.000045
IMP	0.000095	0.003562
indole	0.000093	0.00008
Indole-3-carboxylic acid	0.000012	0.000156
Indoleacrylic acid	0.000126	0.000167
inosine	0.034627	0.041613
isocitrate	0	0.000005

Kynurenic acid	0.000053	0.000039
Kynurenine	0.000012	0.00001
lactate	0.039292	0.02814
L-arginino-succinate	0.000182	0.000217
leucine-isoleucine	0.015203	0.01665
lipoate	0.000002	0.000002
lysine	0.000416	0.000628
malate	0.00667	0.00322
Maleic acid	0.001527	0.000895
malonyl-CoA	0.000014	0.000008
methionine	0.000346	0.000665
Methionine sulfoxide	0.000129	0.000122
Methylcysteine	0.000222	0.000311
Methylmalonic acid	0.011455	0.013216
methylnicotinamide	0.000005	0.000002
myo-inositol	0.039384	0.031384
N1-Methyl-2_2-pyridone-3_5-carboximide	0.000024	0.000052
N1-Methyl-2_4-pyridone-3_5-carboximide	0.000002	0.000001
N6-Acetyl-L-lysine	0.008755	0.022343
N-acetyl spermidine	0.00002	0.000036
N-acetyl spermine	0.000001	0.000005
N-acetyl-glucosamine	0.00001	0.000024
N-acetyl-glucosamine-1-phosphate	0.000228	0.000353
N-acetyl-glutamate	0.000045	0.000074
N-acetyl-glutamine	0.000206	0.000161
N-Acetyl-L-alanine	0.023136	0.020701
N-acetyl-L-ornithine	0	0.000012
N-Acetylputrescine	0	0.000001
NAD+	0.011834	0.006546
NADH	0.000037	0.000075
NADP+	0.000628	0.000747
NADPH	0.000003	0.000005
N-carbamoyl-L-aspartate	0.000007	0.000004
N-carbamoyl-L-aspartate-nega	0.000161	0.000022
Ng,NG-dimethyl-L-arginine	0.004801	0.008792
nicotinamide	0.104521	0.073744
Nicotinamide Riboside	0.000004	0.000003
Nicotinamide ribotide	0.000035	0.000102
nicotinate	0.000049	0.000031
O8P-O1P	0.000021	0.000046
O-acetyl-L-serine	0.000004	0.000004
OBP	0.000083	0.00009
ornithine	0.000141	0.000313

orotate	0.00008	0.000185
orotidine-5-phosphate	0.000005	0.000005
oxaloacetate	0.000041	0.000121
p-aminobenzoate	0.000009	0.000069
pantothenate	0.016441	0.014858
phenylalanine	0.00367	0.004051
Phenyllactic acid	0.00006	0.000079
Phenylpropiolic acid	0.009444	0.010058
phenylpyruvate	0.000001	0.000001
phosphocreatine	0.001143	0.001488
phosphoenolpyruvate	0.000123	0.00026
Phosphorylcholine	0.019645	0.020498
p-hydroxybenzoate	0.00154	0.001701
proline	0.008336	0.012059
propionyl-CoA	0.000023	0
purine	0.000111	0.000114
putrescine	0.000005	0.000013
pyridoxine	0.000003	0
Pyroglutamic acid	0.001301	0.001452
Pyrophosphate	0.002727	0.002675
pyruvate	0.000055	0.000037
quinolinate	0.00014	0.000138
retinoic acid	0.000001	0.000004
riboflavin	0.000019	0.000006
ribose-phosphate	0.002319	0.002213
S-adenosyl-L-homocysteine	0.001661	0.000715
S-adenosyl-L-methionine	0.001553	0.00059
sarcosine	0.013422	0.013175
SBP	0.000059	0.000039
serine	0.000154	0.000462
S-methyl-5-thioadenosine	0.000058	0.000167
sn-glycerol-3-phosphate	0.039892	0.024587
spermidine	0	0.000129
spermine	0.000006	0.000035
S-ribosyl-L-homocysteine	0.000013	0.000008
succinate	0.010996	0.012513
succinyl-CoA-methylmalonyl-CoA	0	0
succinyl-CoA	0.000007	0.000017
taurine	0.054615	0.0216
Taurodeoxycholic acid	0.000025	0.000001
thiamine	0	0.000006
Thiamine pyrophosphate	0.000079	0.000201
thiamine-phosphate	0.00005	0.00004

threonine	0.0001	0.001839
thymidine	0.000006	0.000005
thymine	0	0.000011
trans, trans-farnesyl diphosphate	0.000014	0.000003
trehalose-6-Phosphate	0.000576	0.001087
trehalose-sucrose	0.002011	0.000321
tryptophan	0.000315	0.000396
tyrosine	0.000131	0.00029
UDP-D-glucose	0.001818	0.002173
UDP-D-glucuronate	0.000147	0.000174
UDP-N-acetyl-glucosamine	0.000729	0.000373
UDP	0.000131	0.000869
UMP	0.000017	0.00007
uracil	0.001478	0.002173
Urea	0.002252	0.001923
Uric acid	0.000015	0.00005
uridine	0.001889	0.000837
UTP	0.001203	0.002253
valine	0.000017	0.00005
xanthine	0.000203	0.00035
xanthosine	0.000152	0.000128
xanthosine-5-phosphate	0.00806	0
Xanthurenic acid	0.000199	0.007195
	1	1
