# Supplementary material for: Robust effect of metabolic syndrome on major metabolic pathways in the myocardium
Source: PLoS One. 2019 Dec 2;14(12):e0225857. doi: 10.1371/journal.pone.0225857 (PMC6886832; doi:10.1371/journal.pone.0225857)
Supplement: S2 Table — RNA-Seq reads over multiple runs. Plots show entire transcriptome RNA-Seq reads for LD (Lanes 1–4) across 4 technical replicates and for MetS across 4 replicates (lanes 5–8). (PDF) [file pone.0225857.s004.pdf]

ID	log2FoldChange	pvalue	padj	1	2	3	4	5	6	7	8	Gene.name
ENSSSCG00000024109	0.987709	2.39E-12	1.53E-10	1248.64	581.719	704.379	504.7887	1136.67	1321.867	375.3823	584.79677	BDH1
ENSSSCG00000012411	0.742422	2.79E-08	7.67E-07	4233.7	2182.49	2565.5	1378.378	3455.32	3891.315	1790.937	2175.6484	PHKA1
ENSSSCG00000016863	0.676898	4.87E-08	1.28E-06	41573	24790.6	25998.7	14151.49	35832.2	45602.24	14873.61	26152.316	OXCT1
ENSSSCG00000011698	-0.739	1.45E-07	3.37E-06	7035.32	13461.6	10596.3	2214.978	6608.67	6756.818	2294.675	9994.111	GYG1
ENSSSCG00000012880	-1.02221	2.56E-07	5.6E-06	492.993	1399.24	1328.62	1159.708	845.496	591.0167	1094.663	1191.496	CPT1A
ENSSSCG00000013022	0.631732	1.41E-06	2.51E-05	17829.1	10984.1	12596.6	4857.503	17003.7	16575.78	4803.682	9602.0563	PYGM
ENSSSCG00000022343	-0.51443	0.000271	0.00235	1558.19	2300.91	1852.68	1672.112	1426.77	1281.447	1823.631	1942.0217	ENO1
ENSSSCG00000004456	-0.34508	0.008537	0.03798	559.698	698.063	700.161	419.9319	498.239	568.0752	546.1207	668.02627	PGM3
ENSSSCG00000029944	-0.37173	0.01672	0.0634	455.472	604.573	519.848	504.7887	418.434	377.9885	362.0622	497.18676	FASN
ENSSSCG00000026025	0.233435	0.037167	0.11567	3287.32	2557.49	2720.5	1529.597	3289.24	2959.453	1704.962	2831.9933	HMGCL
ENSSSCG00000010517	-0.26873	0.062069	0.16789	8504.91	8927.32	9314.04	2162.758	6328.28	6938.165	1861.169	7990.0322	PGAM1
ENSSSCG00000012157	0.343984	0.088063	0.21639	509.67	549.517	581.007	1090.082	741.966	892.5336	1215.754	558.51377	PHKA2
ENSSSCG00000004049	-0.7426	0.090825	0.22128	70.8743	76.8701	65.3764	33.7251	19.4119	30.58867	54.49097	60.596918	ACAT2
ENSSSCG00000017421	-0.08835	0.595101	0.75684	316.85	360.458	328.991	236.0757	283.629	314.6263	227.6512	285.46259	ACLY
ENSSSCG00000006340	0.08417	0.607317	0.76624	928.662	751.041	688.562	946.4787	672.946	687.1525	934.8229	719.86219	UAP1
ENSSSCG00000002888	0.007031	0.978915	0.98939	69.832	77.9088	60.1042	75.06556	69.0201	74.28676	123.5129	73.738419	GAPDHS
